# CARMA3: A potential therapeutic target in non-cancer diseases

**DOI:** 10.3389/fimmu.2022.1057980

**Published:** 2022-12-22

**Authors:** Zhen Gui, Yan Zhang, Aihua Zhang, Weiwei Xia, Zhanjun Jia

**Affiliations:** ^1^ Department of Clinical Laboratory, Children’s Hospital of Nanjing Medical University, Nanjing, China; ^2^ Department of Nephrology, Children’s Hospital of Nanjing Medical University, Nanjing, China; ^3^ Jiangsu Key Laboratory of Pediatrics, Nanjing Medical University, Nanjing, China

**Keywords:** CARMA3, BCL10, MALT1, NF-κB, non-cancer diseases

## Abstract

Caspase recruitment domain and membrane-associated guanylate kinase-like protein 3 (CARMA3) is a scaffold protein widely expressed in non-hematopoietic cells. It is encoded by the caspase recruitment domain protein 10 (*CARD10*) gene. CARMA3 can form a CARMA3-BCL10-MALT1 complex by recruiting B cell lymphoma 10 (BCL10) and mucosa-​associated lymphoid tissue lymphoma translocation protein 1 (MALT1), thereby activating nuclear factor-​κB (NF-κB), a key transcription factor that involves in various biological responses. CARMA3 mediates different receptors-dependent signaling pathways, including G protein-coupled receptors (GPCRs) and receptor tyrosine kinases (RTKs). Inappropriate expression and activation of GPCRs and/or RTKs/CARMA3 signaling lead to the pathogenesis of human diseases. Emerging studies have reported that CARMA3 mediates the development of various types of cancers. Moreover, CARMA3 and its partners participate in human non-cancer diseases, including atherogenesis, abdominal aortic aneurysm, asthma, pulmonary fibrosis, liver fibrosis, insulin resistance, inflammatory bowel disease, and psoriasis. Here we provide a review on its structure, regulation, and molecular function, and further highlight recent findings in human non-cancerous diseases, which will provide a novel therapeutic target.

## Introduction

CARMA3 is a member of the CARMA family, which includes CARMA1, CARMA2, and CARMA3 proteins. The three CARMA proteins are encoded by different genes and expressed in different tissues. CARMA1 is encoded by *CARD11* and is mainly expressed in lymphoid and hematopoietic tissues, and CARMA2 is encoded by *CARD14* and expressed in mucosal and skin tissues, while CARMA3 is encoded by *CARD10* and is widely expressed in tissues other than hematopoietic cells (1). Despite different expression patterns, CARMA family members appear to share functional similarity. They are regarded as molecular scaffolds that assist in recruiting and assembling signal transduction molecules. All three CARMA proteins can recruit BCL10 and MALT1 to form the CARMA-BCL10-MALT1 complex (commonly known as CBM), which regulates NF-κB activation and is involved in the occurrence of various diseases (1). CARMA1 is involved in both innate and adaptive immune systems (2) and is crucial for antigen receptor-dependent lymphocyte activation and NF-κB signaling (3–5). Both CARMA1 deficiency and mutations are associated with immunodeficiency (6–8). CARMA1 mutations can also cause atopic diseases, B cell expansion and susceptibility to B cell lymphoma (8–11). CARMA2 is the less-studied CARMA protein family member. CARMA2 mutations are associated with inflammatory skin disorders, especially psoriasis (12). In cancer cells, CARMA2 knockout leads to inhibition of cell proliferation and migration and decrease in cell survival (13, 14). The function of CARMA3 is similar to that of other CARMA proteins, it mediates GPCRs- and RTKs-induced signaling pathways, which are linked to human disease pathogenesis. Despite its potential significance, a discussion on the function of CARMA3 in non-cancer diseases is lacking. Hence, this study aims to review an understanding of the molecular function, mutation, and regulation of CARMA3 and highlight recent findings in non-cancer diseases.

## Structural features of CARMA3

**Figure 1:**
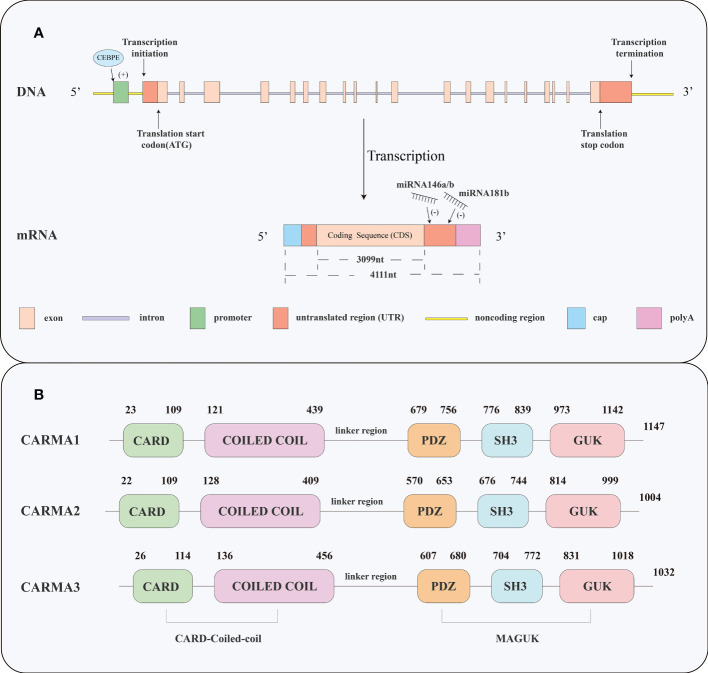
Gene structure of *CARD10* and protein structures of CARMA1, CARMA2, and CARMA3. **(A)** The *CARD10* gene consists of 20 exons. Its mRNA and CDS lengths are 4111 and 3099nt respectively. CEBPE is a transcription factor, that targets the promoter region of *CARD10*. microRNA -146a/b and microRNA-181b regulate *CARD10* by acting on the 3-’UTR of *CARD10* mRNA. **(B)** All the three CARMA proteins are comprised of five functional domains: a CARD domain, a CC domain, a PDZ domain, an SH3 domain, and a GUK domain.

CARMA3, also known as CARD10 or BCL10-interacting membrane protein 1 (Bimp1), was discovered in 2001 (15). The *CARD10* gene is located on human chromosome 22q13.1 and consists of 20 exons. Its mRNA and coding sequence (CDS) lengths are 4111nt and 3099nt respectively. It encodes a 1032-amino acid protein, which comprises five functional domains: a caspase recruitment domain (CARD) domain, a coiled-coil (CC) domain, a PDZ domain, an SH3 domain, and a guanylate kinase-like (GUK) domain (15), as shown in **Figure 1**. The CARD domain is a protein module composed of six or seven antiparallel alpha helices. It is necessary for BCL10 recruitment, which is essential for NF-κB signaling (15). Close to CARD domain is a CC domain, it is composed of one or more alpha-helical peptides that are intertwined in a super-helical fashion (16). CC domain can mediate self-association, leading to the aggregation and activation of BCL10 in response to upstream signals (15, 17). The Membrane-associated GUK (MAGUK) domain comprises a PDZ domain, SH3 domain, and GUK domain (18). The PDZ and SH3 domains of CARMA3 are essential for NF-κB activation, which is not affected by deletion of GUK domain (15).

## Regulation of CARMA3

Although the structure of CARMA3 has been elucidated, the regulation of CARMA3 remains largely unknown. Several microRNAs (miRNAs) reportedly play important roles in regulating CARMA3 levels (**Figure 1A**). miRNA146a is upregulated in the serum and tissues of abdominal aortic aneurysm (AAA) patients and inhibits CARMA3 expression in human umbilical vein endothelial cells (19). It inhibits NF-κB activation by targeting *CARD10* through negative feedback (20), and miR-146a knockdown significantly suppressed lipopolysaccharide-induced cell migration and tube formation (21). Silencing of miR-146a/b that directly targets *CARD10*, resulted in inflammation in psoriatic skin (22). miR-181b has also been identified as an important regulator of arterial thrombosis that directly targets *CARD10* in endothelial cells (ECs). Knockdown of CARMA3 reduces thrombin-induced gene expression and NF-κB signaling, while overexpression can effectively rescue miR-181b-mediated inhibition of NF-κB target genes, such as *VCAM-1*, *ICAM-1*, *and E-SELECTIN* (23).

Additionally, CEBPE, a member of the CCAAT/enhancer binding protein (C/EBP) family of transcription factors, regulates *CARD10* transcription (24), as shown in **Figure 1A**. Chromatin immunoprecipitation-polymerase chain reaction (ChIP-PCR), luciferase reporter assay and electrophoretic mobility shift assay confirmed that CEBPE binds regulatory elements upstream of *CARD10* (-1.5 kb and -7kb). In immature granulocytes from CEBPE-knockout mice, the expression of CARMA3 was significantly lower than that at the same stage of differentiation in wild-type mice (24).

Some studies reported that A20 regulated the NF-κB signaling pathway and downregulated proinflammatory cytokines expression (25–27). A20 negatively regulates CARMA3- and BCL10-mediated NF-κB activation *via* its deubiquitylation activity (28). It directly deubiquitylates IKKγ/NEMO and/or TRAF6, thereby suppressing NF-κB activation by perturbing assembly of the complex comprising CARMA3, BCL10 and IKKγ/NEMO (28).

These results indicate that CARMA3 is regulated at the transcriptional and CBM complex formation level by different regulators. All the CARMA3 regulators reported have been listed in **Table 1**.

**Table 1:** List of CARMA3 regulators.

Regulators	Regulating effect	References
microRNA-146a/b	Transcription inhibition	(19–22)
microRNA-181b	Transcription inhibition	(23)
CEBPE	Transcriptional activation	(24)
A20	NF-κB signaling inhibition	(25–28)

## CARMA3 in GPCRs- and RTKs-induced NF-κB activation

**Figure 2:**
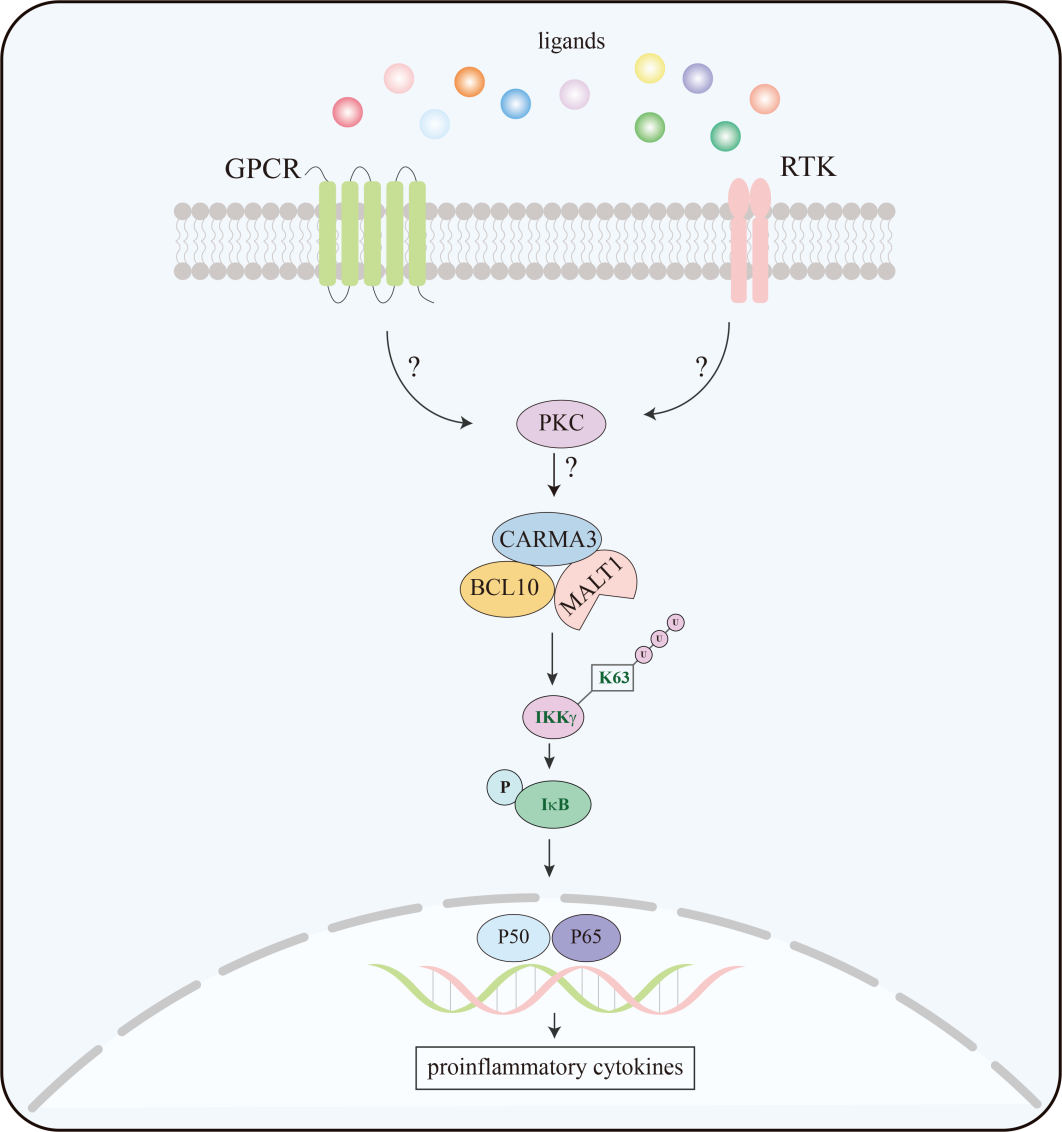
Schematic overview of GPCR/RTK-CARMA3 signaling pathway leading to NF-κB activation. GPCRs and RTKs bind the ligands and activate the downstream signaling pathway. Upon activation, CARMA3 forms a complex with BCL10 and MALT1, resulting in K63-linked ubiquitination of IKKγ. This event activates IKK complex, which triggers NF-κB activation by phosphorylation of IκB.

NF-κB is a transcription factor that involves in cell adhesion, cell proliferation, cell survival, immune responses, and inflammatory responses (29–31). In the dormant state, NF-κB is sequestered in the cytoplasm by interacting with the inhibitor of nuclear factor κB (IκB), which masks the nuclear localization signal of NF-κB. Upon exposure to stimuli, IκB kinase (IKK) complex is activated, leading to phosphorylation and following ubiquitination-mediated degradation of IκB, then NF-κB is released and translocated to the nucleus where it initiates transcription of its target genes (29, 31, 32). The IKK complex comprises three subunits: two protein kinase subunits, IKKα and IKKβ (or IKKl and IKK2), and an essential regulatory subunit, IKKγ (also known as NF-κB essential modulator, NEMO) (32, 33). The phosphorylation of IKKα/β and K63-linked polyubiquitination of NEMO is indispensable when activating the IKK complex (32, 34, 35). CARMA3 is a scaffold molecule involved in the activation of NF-κB through ubiquitination of NEMO (36). Upon stimulation, CARMA3, through its N-terminal effector CARD domain, recruits two downstream signaling molecules, BCL10 and MALT1, to form a CBM complex (37), which participates in GPCRs- and RTKs-induced NF-κB signaling pathways (**Figure 2**).

GPCRs are transmembrane receptors and play a vital role in various cell process signaling activated by a variety of ligands (38). Several ligands, such as thrombin, CXCL8/IL8, lysophosphatidic acid (LPA), and angiotensin II (Ang II), have been shown to induce NF-κB activation (36, 39–43). GPCR-induced NF-κB activation is defective in CARMA3-deficient mouse embryonic fibroblasts (MEFs), indicating that CARMA3 is required for GPCR-induced NF-κB signaling pathway (39). In vascular cells, angiotensin II type I receptor (AGTR1), a member of the GPCR superfamily, induces the activation of NF-κB by triggering the CBM signalosome, leading to vascular disease (43). Additionally, the CBM complex is essential for the activation of Ang II-induced NF-κB in hepatocytes. It is responsible for transmitting the signal from AGTR1 to the IKK complex and activating NF-κB through ubiquitination of NEMO (36). However, how CARMA3 connects to GPCRs and regulates NF-κB signaling pathway remains elusive. Sun and Lin demonstrated that CARMA3 can form a complex with β-arrestin 2, which functions as a positive regulator by recruiting CARMA3 to LPA receptors, leading to NF-κB activation (40). In addition, a DEP domain-containing protein, DEPDC7, was identified as a CARMA3-binding partner (44), which could be a bridge linking activated GPCRs to the CBM signalosome.

RTKs constitute a class of transmembrane receptors that are involved in mediating various cellular responses. Epidermal growth factor (EGF) and vascular endothelial growth factor (VEGF) are known to induce NF-κB activation (31, 45, 46). In MEFs, knockdown of CARMA3 impairs the activation of the IKK complex, resulting in a defect in EGFR-induced IκBα phosphorylation and NF-κB activation, which indicates that CARMA3 is essential to the activation of EGFR-induced NF-κB (47). Besides, CBM complex plays a key role in cell proliferation, migration, and invasion (47, 48). To define the mechanism of EGFR-induced NF-κB activation, Jiang and Lin performed high-throughput screening and successfully identified that TMEM43 could bind to CARMA3 and act as a linker molecule connecting EGFR to CARMA3 and the CARMA3-containing signaling complex, activating the IKK complex (45). Altogether, these studies reveal that CARMA3 is essential for GPCR- and RTK-induced NF-κB activation.

## CARMA3 in viral infections and DNA damage-induced NF-κB activation

CARMA3 is also involved in viral infections and DNA damage. CARMA3 contributes to antiviral response by regulating RIG-I/MAVS-induced IRF3 and NF-κB activation. Upon RIG-I activation, MAVS is activated in the early stages of viral infection, activating IKKα/IKKβ/NEMO in a CARMA3-dependent manner. CARMA3 binds to MAVS *via* the C-terminal GUK domain and blocks downstream activation of IRF3 by preventing the formation of MAVS oligomerization. In the late stages of virus infection, CARMA3 is degraded by the proteasome, releasing MAVS to form high-molecular-weight aggregates and activate IRF3 (49). Moreover, CARMA3 mediates RIG-I/MAVS-induced NF-κB activation. In MEF cells, knockout of CARMA3 partially inhibited NF-κB activation following vesicular stomatitis virus (VSV) infection. When attacked by VSV or influenza A virus (IAV), CARMA3-deficient mice showed milder disease symptoms than wild-type (WT) mice because of less inflammatory response and stronger ability to clear the infected virus, indicating that CARMA3 plays a negative role in the antiviral innate immune response (MAVS-induced IRF3 activation) but a positive role in the pro-inflammatory response (MAVS-induced NF-κB activation) (49).

CARMA3 mediates DNA damage-induced NF-κB signaling pathway by recruiting TRAF6 in a PKC-independent mechanism (50). After doxorubicin treatment, the number of dead cells in CARMA3-deficient MEFs was 2-fold than that of WT control cells, indicating that CARMA3 is essential to DNA damage-induced apoptosis. To investigate the role of CARMA3 in DNA damage, Zhao et al. monitored the survival of WT and CARMA3-deficient mice after 12 Gy irradiation. All WT mice survived within 14 days after irradiation, whereas approximately 23% of CARMA3 knockout mice died, suggesting that CARMA3 partially protects mice from the effects of irradiation. Furthermore, CARMA3 knockout mice had impaired tissue repair and cell proliferation after irradiation, indicating that CARMA3 plays a protective role in cell survival (50). This study suggests that CARMA3 plays a vital role in DNA damage-induced NF-κB signaling pathway and provides a molecular target.

## CARMA3 in cancers

CARMA3, an oncoprotein overexpressed in various cancers, participates in cancer cell proliferation, migration, invasion, and apoptosis; it is related to the tumor node metastasis (TNM) stage, tumor status and grade, metastasis, and poor prognosis (51–59). CARMA3 has similar function in various cancers. In bladder cancer, CARMA3 expression is associated with tumor status and grade. CARMA3 knockdown leads to decrease in cell proliferation and NF-κB signaling (51), and to inhibition of cell migration and invasion *via* blocking β-catenin signaling pathway (52). In lung cancer, CARMA3 is overexpressed and related to the TNM stage and tumor status. CARMA3 deficiency leads to inhibition of tumor cell proliferation and invasion, and to cell cycle arrest at the G1/S boundary (53). Moreover, CARMA3 promotes cell invasion and migration, and is positively correlated with lung cancer stemness, metastasis, and poor survival outcomes (54). Similar results have been observed in hepatocellular carcinoma (56), colorectal cancer (57, 58), ovarian cancer (59), breast cancer (60, 61), and renal cell carcinoma (62–64). In conclusion, CARMA3 is involved in the development and pathogenesis of various types of cancers. These findings suggest that CARMA3 would be a promising therapeutic target for cancer.

## CARMA3 in non-cancer diseases

Over the past decades, a lot of studies have indicated the importance of CARMA3 in non-cancer diseases. Here, we illustrate the role of CARMA3 in non-tumor diseases from two aspects: CBM complex and CARMA3 mutations.

## CBM complex in non-cancer diseases

### CBM complex in cardiovascular diseases

Inflammation is a key characteristic in the pathogenesis of cardiovascular diseases, including atherosclerosis, hypertension, and abdominal aortic aneurysms (65). In the inflammatory process, blood vessels and perivascular connective tissue are vital regulators, and congestion and increased capillary permeability are important features of inflamed tissues (66). In murine endothelial cells, CARMA3 assembles BCL10 and MALT1 to form a CBM signalosome, which triggers the activation of NF-κB induced by CXCL8/IL8, an essential chemokine involved in pro-angiogenic and pro-inflammatory processes. This process controls VEGF expression and promotes the autocrine activation of VEGF receptors (46). The CBM complex also mediates the activation of thrombin-induced NF-κB in murine endothelial cells, and CARMA3 deficiency completely blocks the thrombin-induced phosphorylation of IκB (41). Using siRNA to disrupt the CBM signalosome, the thrombin-responsive induction of adhesion molecules intercellular adhesion molecule 1 (ICAM1) and vascular cell adhesion molecule 1 (VCAM1), which are relevant to atherogenesis, are dramatically downregulated. In addition, thrombin-induced monocyte/endothelial adhesion was also reduced. With this, the CBM complex plays a key role in proximal steps of NF-κB activation as well as in downstream regulation of NF-κB target genes (41). Moreover, the CBM complex triggers the Ang II-dependent NF-κB inflammatory signaling cascade in endothelial and vascular smooth muscle cells, and then induces pro-inflammatory signals in the vessel wall, which is a vital factor of atherogenesis (43). BCL10 and MALT1 are also important in the activation of Ang II-induced NF-κB, using siRNA to knockdown BCL10 or MALT1, Ang II-induced phosphorylation of IκB is completely ablated. In BCL10-deficient mice, the extent of atherosclerosis is significantly reduced, and the incidence and severity of abdominal aortic aneurysms are significantly lower than those observed in the presence of BCL10 (43). In addition, several pro-inflammatory mediators, which are associated with atherogenesis, such as the SCF/c-Kit ligand, CD40 ligand, CXCL6, IL-1α, and Eotaxin/CCL11, have lower serum levels in BCL10-deficient mice (43). These results indicate that BCL10-deficient mice are dramatically protected against Ang II-induced atherosclerosis and abdominal aortic aneurysms. In addition, MALT1 protease activity is also critical in vascular inflammation, it mediates thrombin-induced endothelial permeability and the activation of MALT1 protease disrupts endothelial capillary tubes (67). These studies indicated the importance of CARMA3 and its binding partners in cardiovascular diseases.

### CBM complex in pulmonary diseases

Asthma is broadly defined as airway inflammation implicated in hyperresponsiveness and mucus hypersecretion. NF-κB plays a key role in the pathogenesis of asthma, including airway epithelial barrier dysfunction, airway hyperreactivity and remodeling, chemokine and cytokine production (68–71). CARMA3 is highly expressed in human airway epithelial cells and mediates LPA-induced NF-κB activation (72). It is necessary for the production of pro-inflammatory cytokines and chemokines, such as CC-​chemokine ligand 20 (CCL20)/macrophage inflammatory protein (MIP)-3α, granulocyte-macrophage colony-stimulating factor (GM-CSF), thymic stromal lymphopoietin (TSLP), IL-8, IL-25, and IL-33, which are significantly upregulated in patients with allergic pulmonary inflammation or asthma in response to inhaled stimuli (72–74). In addition, in an asthma mouse model induced by ovalbumin, deletion of CARMA3 in airway epithelial cells (AECs) alleviated allergic airway inflammation (73). The CARMA3-deficient murine model not only attenuated the production of pro-inflammatory cytokines and chemokines, but also impaired dendritic cell (DC) recruitment, DC maturation, DC migration, Ag processing, and resultant T cell proliferation (73, 74). After CARMA3-deficient mice were stimulated with *Alternaria alternata*, the total number and percentage of type 2 innate lymphoid cells (ILC2) in the lungs were reduced, suggesting that CARMA3 mediates the accumulation of ILC2 in the lungs after allergen exposure, which may be one mechanism by which type 2 cytokine production is attenuated in the lung and airway inflammation in CARMA3-deficient mice (74). Additionally, CARMA3 plays a vital role in pulmonary fibrosis (75). Knockdown of CARMA3 attenuates bleomycin-induced inflammation and fibrosis and protects the lung from inflammation, fibrosis, collagen fibril deposition, alveolar epithelial cell destruction, and pathological airway remodeling (75). In conclusion, CARMA3 is recognized as a pro-inflammatory molecule involved in lung inflammation, such as asthma and pulmonary fibrosis.

### CBM complex in hepatic diseases

In hepatocytes, palmitate metabolism stimulates the CBM complex to trigger NF-κB-mediated inflammation in the absence of receptor engagement. siRNA-mediated depletion of CARMA3 effectively blocked palmitate-dependent IκBα phosphorylation. Free fatty acids (FFA), which are essential for the pathogenesis of insulin resistance, are increased in the serum of obese people. Deletion of BCL10 in mice eliminates hepatic NF-κB activation induced by a high-fat diet, resulting in mitigated inflammation and protection from insulin resistance (76).

Ang II is involved in tissue-repairing process by mediating inflammatory cell infiltration, myofibroblast proliferation, and collagen synthesis after liver injury (77). But in chronic liver injury, Ang II induces pathologic liver fibrosis by activating NF-κB (36). Knockout of CARMA3 effectively disrupts the activation of Ang II-induced NF-κB signaling pathway, preventing the occurrence of liver fibrosis (36). However, in an autoimmune hepatitis model induced by Con A, deletion of CARMA3 leads to aggravated liver injury and liver sinusoidal endothelial cells (LSECs) damage, indicating that CARMA3 plays a protective role in the process, which is different from previous studies. CARMA3 exerts its protective effect by preserving mitochondrial integrity, but the detailed mechanisms is still unknown (78). These results indicate that CARMA3 has diverse characters in different hepatic diseases.

### CBM complex in other non-cancer diseases

CARMA3 is widely expressed in various tissues such as the heart, lung, liver, intestine, and kidney (29). However, current research on CARMA3 in non-cancer diseases mainly focuses on the cardiovascular system and lung diseases, with relatively few studies on diseases of other systems. Herein, we briefly overview the role of CARMA3 in other disorders.

In intestinal epithelial cells (IECs), CBM signalosomes activate the NF-κB signaling pathway triggered by platelet-activating factor (PAF) and play a pivotal role in IL-8 production (79). Subsequently, Dudeja et al. demonstrated that *Lactobacillus acidophilus*, a probiotic, could eliminate PAF-induced inflammation in IECs by blocking PAF-induced NF-κB activation and IL-8 production. In addition*, Lactobacillus acidophilus* culture supernatant (LA-CS) can attenuate the PAF-induced elevation of BCL10 mRNA and protein levels, BCL10 promoter activity, BCL10 interaction with MALT1, and PAF-induced ubiquitination of IKKγ (80). These results suggest a novel target complex for the treatment of PAF-induced inflammation in IECs.

In normal human epidermal keratinocytes (NHEKs), CARMA2 is more investigated, it is expressed in keratinocytes and its mutations increase the risk of inflammatory skin disease (81). Recently, a CARMA3-dependent tonic signalosome was described, which activates MALT1 protease activity and mediates IL-17/TNF-α−induced keratinocyte inflammatory response (82). In proliferating NHEKs, the level of CARMA3 is 10-fold higher than that of CARMA2. During differentiation, CARMA2 was upregulated and CARMA3 was downregulated, indicating that CARMA3 may be involved in keratinocytes, particularly during their proliferation (82). Subsequently, knockdown of MALT1 or CARMA3 in proliferating NHEKs abrogated the cleavage of MALT1 substrates, indicating that CARMA3 is fundamental to MALT1 protease activity (82). In addition, knockout of CARMA3 led to downregulation of serpin family B member 2 (SERPINB2) at both the mRNA and protein levels, which is positively related to psoriasis severity (22). These results suggest the importance of CARMA3 in psoriasis.

## CARMA3 mutations in non-cancer diseases

Over the past few years, mutations in *CARD11* and *CARD14* have been studied extensively. *CARD11* mutations are linked to lymphoproliferative disorders and immunodeficiencies, whereas *CARD14* variants are associated with several inflammatory skin disorders (83, 84). However, the consequences of *CARD10* mutations in humans remain unclear. De Boer et al. previously conducted a genome-wide survey and first identified the *CARD10* polymorphism rs6000782 as a likely risk factor for type1 autoimmune hepatitis (AIH) in Dutch and German populations (85), which Motawi et al. also concluded with a cohort of Egyptian children (86). However, these results could not be replicated in a Japanese population (87). This discrepancy may result from several factors: (1) ethnic variations, (2) different sample sizes, and (3) different experimental conditions and methods. In addition, *CARD10* rs9607469 and *CARD10* rs9610775 are associated with primary open-angle glaucoma and amnestic mild cognitive impairment, respectively (88–90). Apart from *CARD10* polymorphism, Hang et al. were the first to identify two in-frame deletions in *CARD10* among 101 cases of ovarian endometriosis, which may cause the disease (91). Luo et al. used exome sequencing to identify the causative genes of two siblings, who are affected by autoimmune deficiency from a consanguineous family. They identified a new homozygous missense mutation (c.1258C>T; p. R420C) of *CARD10*, which is implicated in rare diseases, repeated infections, Crohn’s disease, allergic diseases, and other disorders (92). Among the above mentioned *CARD10* mutations, rs9610775 and R420C are missense variants and are located in exons 4 and 7 of *CARD10* respectively. Both mutations alter the CARMA3 CC domain, which is associated with BCL10 aggregation and activation, suggesting that the rs9610775 and R420C mutations affect CBM assembly and the subsequent NF-κB activation (90–93). To date, no study has reported the direct effect of *CARD10* mutations on NF-κB signaling. However, Said et al. found that the NF-κB p65 level is increase in the AIH patients with *CARD10* polymorphism rs6000782, suggesting that the *CARD10* polymorphism rs6000782 may cause disease by affecting NF-κB signaling and the expression levels of downstream genes (86). Further studies should be conducted to elucidate the exact effect of *CARD10* mutations on the NF-κB signaling pathway.

**Figure 3:**
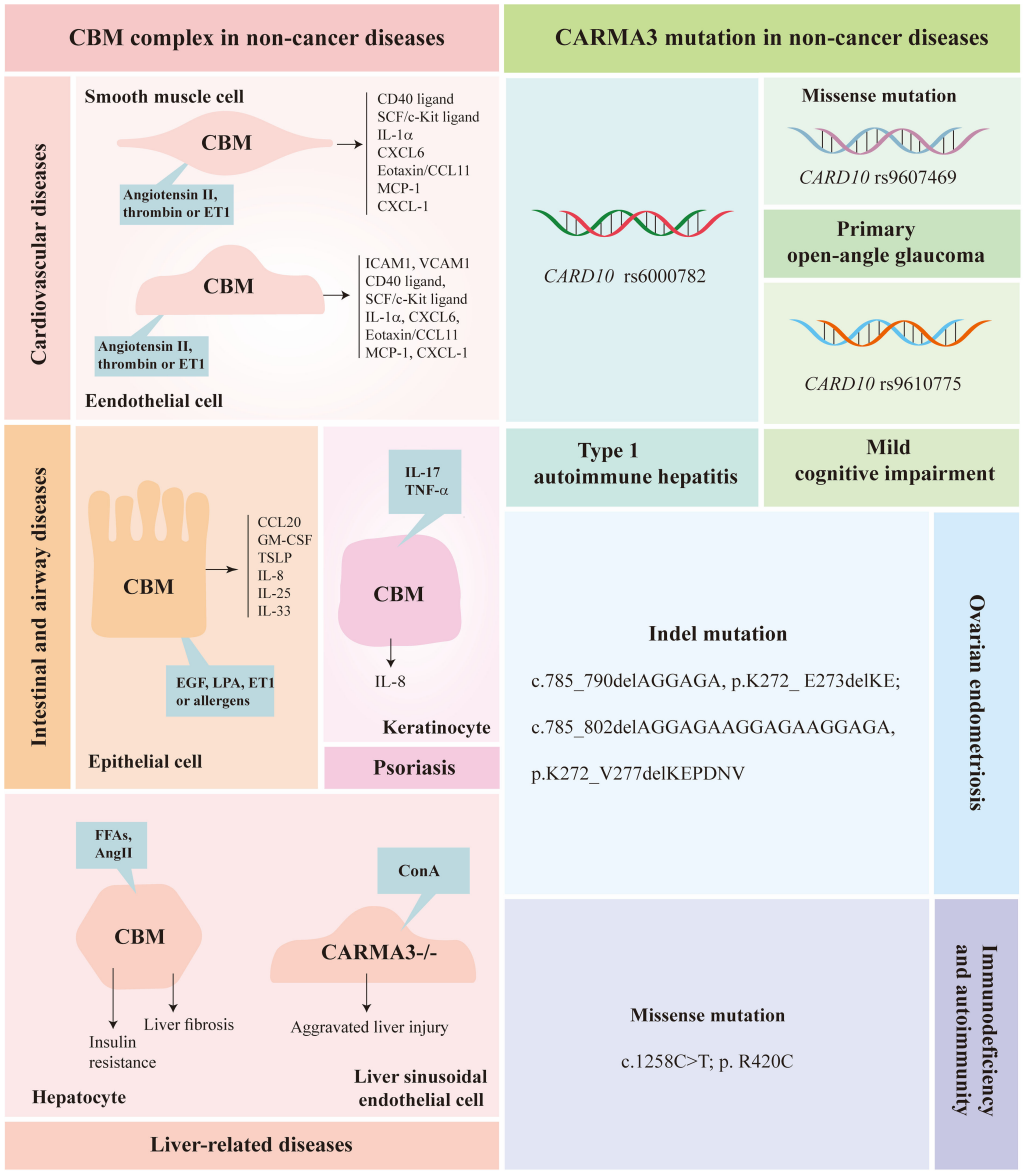
Overview of CARMA3 in non-cancer diseases. CBM complex are implicated in the pathogenesis of cardiovascular diseases, pulmonary diseases, liver-related diseases, intestinal disease and psoriasis; *carma3* mutations are related to type1 autoimmune hepatitis, primary open-angle glaucoma, mild cognitive impairment, ovarian endometriosis and immunodeficiency and autoimmunity.

All the functions of CARMA3 in non-cancer diseases were summarized in **Figure 3**.

## Conclusions and perspectives

CARMA3 is widely expressed in a variety of tissues (29) and plays key roles in the pathological process of human diseases (37). To date, most of the existing literature on the role of CARMA3 in diseases has focused on a variety of cancers. Relatively fewer studies investigated the role of CARMA3 in non-cancer diseases. In addition, most studies focused on airway epithelia and the cardiovascular system while searching in the literature for association between non-cancer disease and CARMA3. Considering that CARMA3 affects cell inflammation, proliferation, survival, migration, and other cell processes (55, 58, 64, 94), it should be taken into account that CARMA3 could be involved in other non-cancer diseases, such as urinary system diseases, nervous system diseases, and bone and joint disorders. Further studies exploring these areas are needed.

Current studies on the regulation of CARMA3 mainly focus on the transcriptional level, with no investigations on phosphorylation, ubiquitination, and acetylation of CARMA3 proteins. To further understand the contribution of CARMA3 in diseases, its regulation at the protein level is worthy of investigation. Furthermore, CARMA3-dependent signal transmission plays a fundamental role in pathophysiology of various diseases, indicating that generating molecular tools, such as novel inhibitors specific to CARMA3 and small peptides target to CBM signalosome, would be immeasurable in treating CARMA3-related disorders.

## Author contributions

WX and ZJ conducted the review, and ZG, WX, and YZ drafted the manuscript. All the authors have written, reviewed and approved the manuscript.
